# Exogenous Addition of a C-Xylopyranoside Derivative Stimulates Keratinocyte Dermatan Sulfate Synthesis and Promotes Migration

**DOI:** 10.1371/journal.pone.0025480

**Published:** 2011-10-05

**Authors:** Jun Muto, Nandita Natasha Naidu, Kenshi Yamasaki, Nathalie Pineau, Lionel Breton, Richard L. Gallo

**Affiliations:** 1 Division of Dermatology, University of California, San Diego and Veterans Affairs San Diego Health Care System, San Diego, California, United States of America; 2 Glycotechnology Core Resource, University of California, San Diego, California, United States of America; 3 L'Oréal Recherche, Clichy, France; Ludwig-Maximilian-University, Germany

## Abstract

As C-Xyloside has been suggested to be an initiator of glycosaminoglycan (GAG) synthesis, and GAGs such as Dermatan sulfate (DS) are potent enhancers of fibroblast growth factor (FGF) - 10 action, we investigated if a C-Xylopyranoside derivative, (C-β-D-xylopyranoside-2-hydroxy-propane, C-Xyloside), could promote DS production by cultured normal human keratinocytes, how this occurs and if C-Xyloside could also stimulate FGF-dependent cell migration and proliferation. C-Xyloside-treated keratinocytes greatly increased secretion of total sulfated GAGs. Majority of the induced GAG was chondroitin sulfate/dermatan sulfate (CS/DS) of which the major secreted GAG was DS. Cells lacking xylosyltransferase enzymatic activity demonstrated that C-Xyloside was able to stimulate GAG synthesis without addition to core proteins. Consistent with the observed increase in DS, keratinocytes treated with C-Xyloside showed enhanced migration in response to FGF-10 and secreted into their culture media GAGs that promoted FGF-10-dependent cellular proliferation. These results indicate that C-Xyloside may enhance epithelial repair by serving as an initiator of DS synthesis.

## Introduction

Proteoglycans (PG) and their glycosaminoglycan (GAG) chains are essential factors in skin growth and development and are thought to act during wound repair to influence growth factor functions [1]. Dermatan sulfate (DS) is the most common GAG in the skin and is estimated to comprise between 36 and 78% of the sulfated GAG in wound fluid [1]. GAGs, such as DS, and their PG core proteins, participate in a variety of functions during wound healing including binding multiple growth factors and promoting their activities [1], [2], [3], [4], [5], [6], [7], [8]. Of these activities, members of the fibroblast growth factor (FGF) family, including FGF-7 and FGF-10, depend on GAGs to participate in important wound repair events including inflammation, repair and regeneration [9] [10]. Because of the important role of GAGs in skin biology, mechanisms to control production or breakdown are potentially clinically important.

Of the several GAGs in wounds, DS has been shown to be a potent enhancer of the action of FGF-7 and -10, and is vital for wound repair [11]. DS consists of repeating disaccharide units of N-acetylgalactosamine and glucuronic acid (GlcA)-linked β1→4 and β1→3, respectively. Similar to modifications that occur in heparin and heparan sulfate (HS), but unlike the other chondroitin sulfates (CS), the GlcA of DS undergoes epimerization of the C-5 carbon, resulting in iduronic acid (IdoA). These structural characteristics of DS make it unique since it resembles some elements of the well studied HS, but yet is considered a CS.

Xylose (Xyl) is a unique component of PGs and is the first sugar attached to the nascent PG core protein when the posttranslational assembly of GAG side chains is initiated. During transport through the secretory pathway, GAG chains are polymerized on the common linkage region GlcUA-Gal-Gal-Xyl-protein. This saccharide serves as a primer for either chondroitin sulfate (CS) type or heparan sulfate (HS) type GAG chain synthesis. Biosynthesis of GAG chains can also be accomplished in an alternative manner, in the absence of core proteins, by using xylosides as primers. β-Xylosides initiate GAG synthesis by serving as acceptors in the first galactosylation step [12]. The type of GAG produced using this technique depends on the structure of the aglycone.

In the present study, we set out to determine if increased GAG synthesis could be stimulated in keratinocytes and to test the hypothesis that C-Xyloside could do this by serving as an initiator of GAG synthesis and thus improve FGF-10-mediated responses.

## Results

### C-Xyloside induces the release of sulfated GAGs from keratinocytes

**Figure 1:**
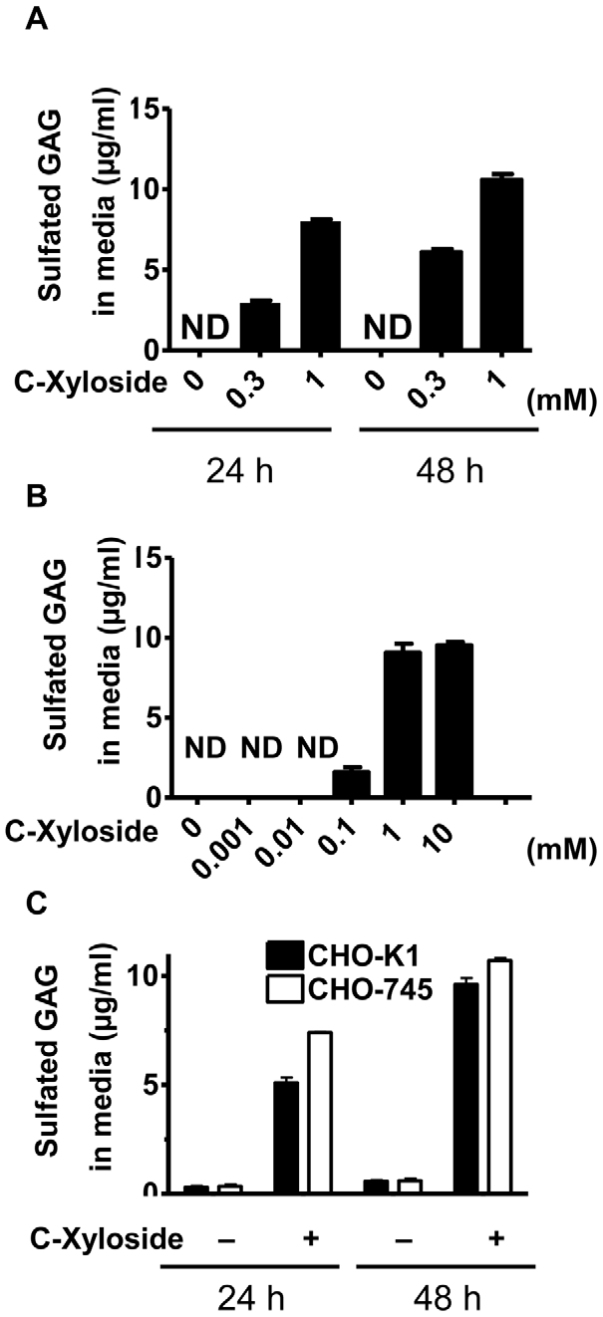
C-Xyloside induces sulfated GAGs from NHEK in culture media without the involvement of Xylosyltransferase. Primary keratinocytes were stimulated with PBS (untreated cells) or C-Xyloside (0.3 mM or 1 mM). (A) Cultured media were collected after 24 and 48 hours. Total sulfated GAG was determined by Blyscan assay. (B), Increasing concentrations of C-Xyloside were added to keratinocytes. Culture media was collected after 24 hours and total sulfated GAG was determined by Blyscan assay. (C), CHO-K1 (black bars) and CHO-745 (white bars) cells were cultured in the presence or absence of C-Xyloside (1 mM) for 24 hours and the amount of sulfated GAG in the media was determined by Blyscan assay. Data are representative of three individual experiments, and presented as mean ± SEM (n = 3). N.D., not detected.

To investigate how C-Xyloside might work on the skin, we studied if it could serve as an initiator of GAG synthesis by cultured normal human epidermal keratinocytes (NHEK). Cells were treated with or without C-Xyloside and the amount of sulfated GAGs in the media and cell lysates measured. A time-dependent increase in sulfated GAGs was observed in the culture media of NHEK, with a mean value of 8.0 µg/ml 24 hours after 1 mM C-Xyloside treatment, and 10.6 µg/ml 48 hours after 1 mM C-Xyloside treatment (Figure 1A). The concentration of sulfated GAGs in cell lysates was also measured after 0.3 mM and 1 mM C-Xyloside treatment, and normalized to cell number. The concentrations in cell lysates after C-Xyloside treatments were within the normal range set by vehicle-treated control cells (data not shown). Treatment of NHEK with doses of C-Xyloside ranging from 0.1 to 10 mM increased the amount of sulfated GAGs in culture media but sulfated GAGs were not detectable in the media of keratinocytes treated at a concentration lower than 10 µM (Figure 1B). In contrast, treatment of 12F fibroblasts with 1 mM C-Xyloside showed that the amount of secreted GAGs in the media from fibroblasts was much less than the amount of GAGs from NHEK culture media after treatment (data not shown). These findings demonstrated that keratinocytes treated with C-Xyloside are stimulated to secrete and increase the abundance of sulfated GAGs in the media.

### C-Xyloside induces xylosyltransferase-deficient mutant CHO-745 cells to synthesize and secrete sulfated GAGs

To determine if the GAG produced by treatment with C-Xyloside was free GAG or GAG associated with a proteoglycan core, the involvement of the xylosyltransferase enzyme in synthesizing and secreting sulfated GAGs was examined by using CHO-745 cells, a xylosyltransferase-deficient mutant CHO cell line that has less than 5% of the xylosyltransferase activity found in wild-type CHO-K1 cells [13]. Xylosyltransferase is necessary for GAG formation on core protein of proteoglycaan, therefore new GAG synthesis observed in these cells would indicate C-Xyloside was serving as the accepter of GAG chain elongation and thus substituting for core protein. CHO-K1 cells were treated with C-Xyloside and incubated for 24 and 48 hours, and total sulfated GAGs were determined. CHO-745 cells produced slightly more total sulfated GAGs compared to CHO-K1 cells (Figure 1C). Thus, sulfated GAG synthesis induced by C-Xyloside was independent of xylosyltransferase, suggesting that the majority of GAGs produced after C-Xyloside stimulation were free GAGs synthesized without core proteins. Supporting this conclusion, C-Xyloside also did not increase mRNA expression of enzymes involved in CS synthesis and secretion of sulfated proteoglycans such as: CS synthase 1 (*CSS1*), *CSS2*, *CSS3*,chondroitin sulfate glucuronyltransferase(*CSGlcAT*), chondroitin sulfate N-acetylgalactosaminyltransferase1(*CSGalNAcT1*) *and CSGalNAcT2* (data not shown).

### Analysis of GAGs secreted from NHEK after treatment with C-Xyloside

The amount, composition, and sulfation patterns of GAGs are essential variables that influence cellular behaviors during development, coagulation, and wound repair [14]. We therefore sought to characterize the sulfated GAGs produced by NHEK treated with C-Xyloside. First, conditioned culture media containing secreted GAG from treated keratinocytes was treated with nitrous acid (to fragment HS) or chondroitinase ABC (to digest CS/DS). Most of total GAGs were digested by chondroitinase ABC, but none by nitrous acid, demonstrating that the majority of the sulfated GAG produced by NHEK treated with C-Xyloside was CS, not HS.

**Figure 2:**
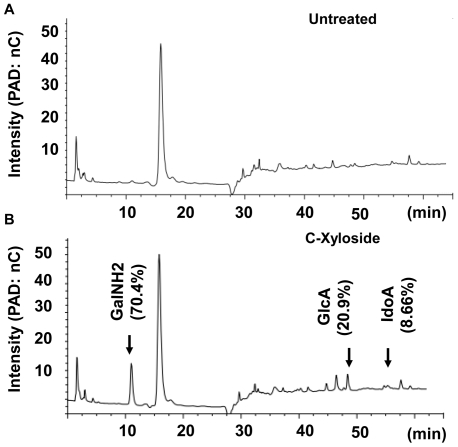
Monosaccharide composition analysis of GAGs release by NHEKs. Media were harvested after 24 hours of culture (A) without or (B) with C-Xyloside (1 mM) stimulation and monosaccharide compositions were analyzed by HPAEC chromatograph as described under ‘materials and methods’. The peak at 17 min may represent a monosaccharide such as glucose in the media (Glc). Arrows indicate the retention time for galactosamine (GalNH2), glucuronic acid (GlcA), and iduronic acid (IdoA). PAD: pulsed amperometric detection; nC: nano-coulomb. Data are representative of three individual experiments.

**Figure 3:**
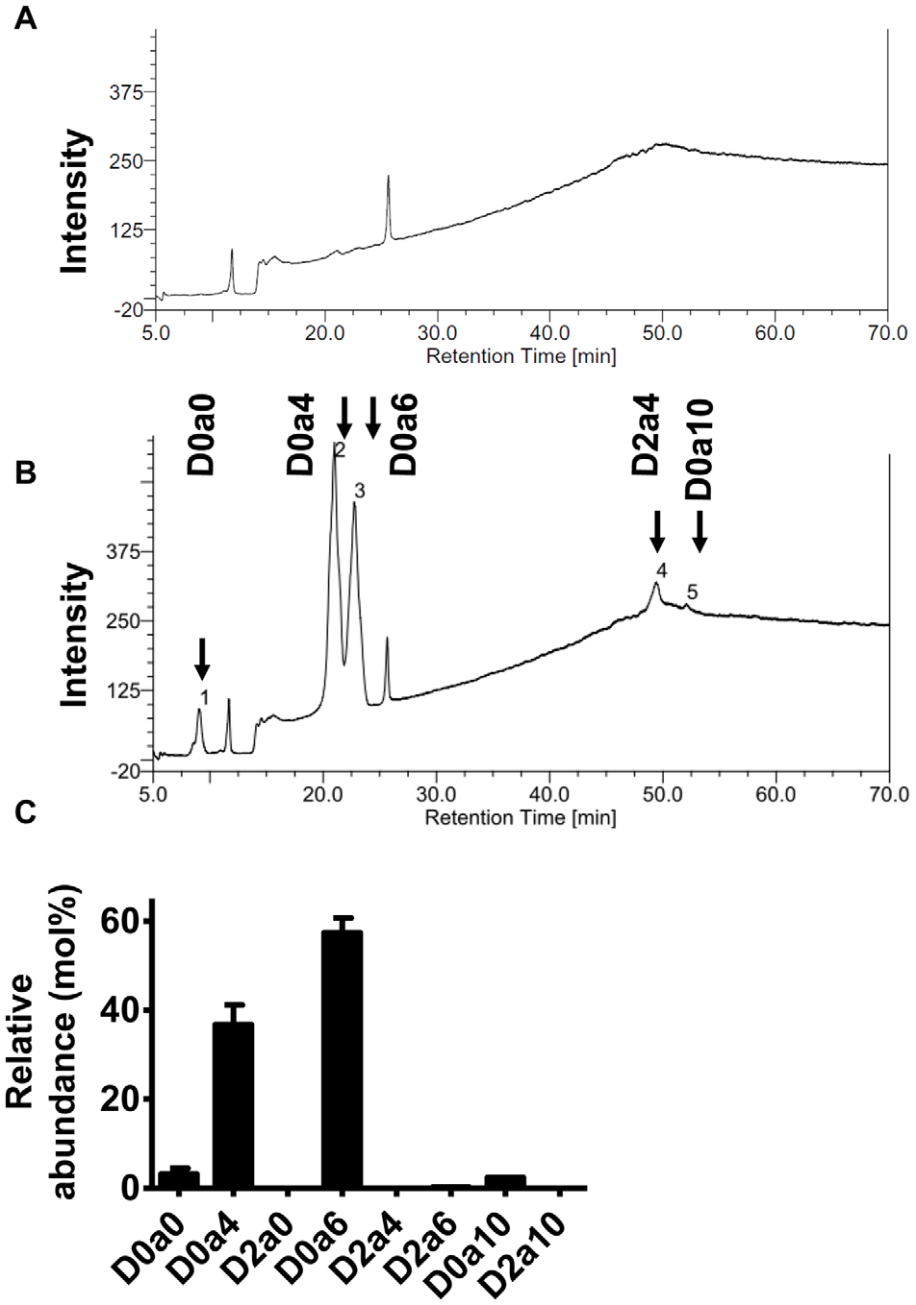
Disaccharide analysis of synthesized GAGs. Media were harvested after 24 hours of culture (A) without or (B) with C-Xyloside (1 mM) stimulation. GAG in culture media were digested with chondroitinase ABC and the disaccharides were resolved by anion-exhange HPLC with post-column derivitization and fluorescence detection (see materials and methods.) Values are expressed as molar percentage. Data are representative of three individual experiments. (C), the disaccharides were resolved by Glycan reductive isotope labeling/mass spectrometry (n = 2). Data are presented as mean ± range. D0a0, ΔUA-GalNAc; D0a4, ΔUA-GalNAc4S; D2a0, ΔUA2S-GalNAc; D0a6, ΔUA-GalNAc6S; D2a4, ΔUA2S-GalNAc4S; D2a6, ΔUA2S-GalNAc6S; D0a10, ΔUA-GalNAc4S6S; D2a10, ΔUA2S-GalNAc4S6S. ΔUA = 4, 5-unsaturated uronic acid.

Analysis of the monosaccharide composition of the GAGs in the conditioned culture media revealed that GAGs produced by NHEK had 70.4% GalNH_2_, 20.9% GlcA and 8.69% IdoA, which is compatible with DS (CS-B) (Figure 2). Analysis of these same samples by Mass Spectrometry confirmed the existence of IdoA, a unique component only found in DS or HS. Analysis of disaccharide composition by using reverse phase ion pair chromatography, revealed that most of the secreted GAGs were sulfated (Figure 3A, B). Quantification of disaccharides was achieved using an aniline tagging method and mass spectrometry as described [15]. D0a4 was 36.7%, D0a6 was 57.4%, and D0a10 was 2.42% (Figure 3C) [16]. Thus, these data taken together show c-Xyloside induces NHEK to produce a sulfated GAG that is predominantly DS.

### C-Xyloside improves the capacity of FGF-10 to promote keratinocyte migration

**Figure 4:**
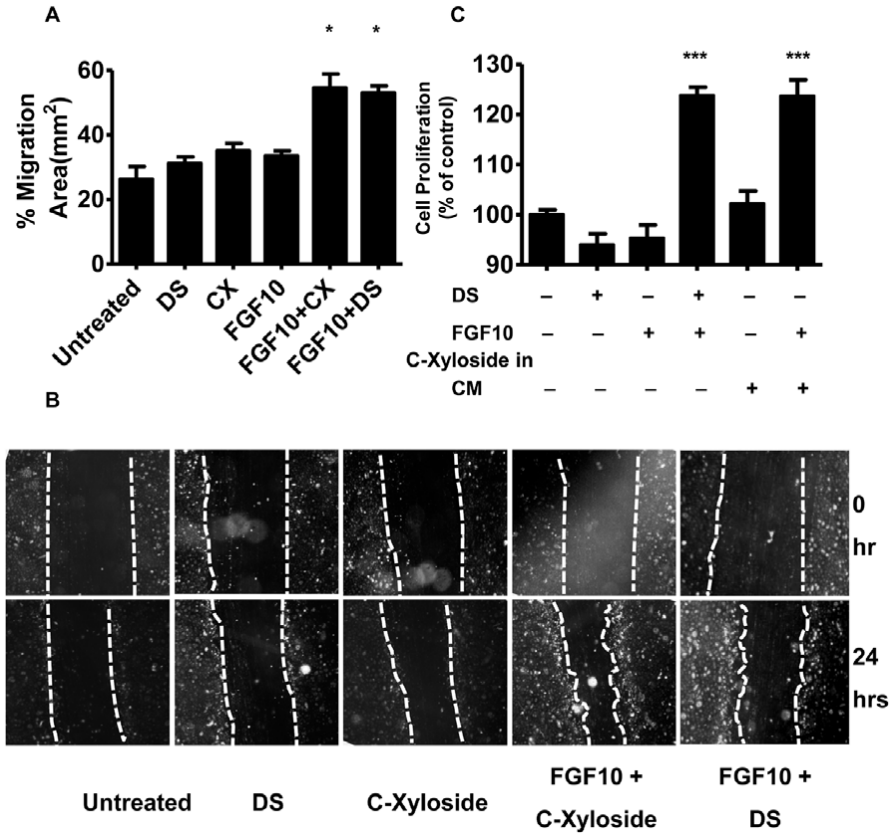
C-Xyloside promotes FGF-10 dependent keratinocyte migration and cell proliferation. (A), Primary keratinocytes were stimulated with DS alone, C-Xyloside (CX) alone, FGF-10 alone, C-Xyloside and FGF-10 combined, DS and FGF-10 combined or vehicle. Data are plotted as the mean ± SEM of three individual experiments with samples in quadruplicate. *, p<0.05 (B), Identical fields were photographed 0 and 24 hours after wounding. (C), Primary keratinocytes were treated with PBS or C-Xyloside in RPMI1640 media. All dilutions were done in this keratinocyte-conditioned medium (CM). Cells were incubated for 3 days at 37°C before performing a nonradioactive cell proliferation assay. Data are represented as the mean ± SEM of triplicate determinations. ***, p<0.001.

Given that C-Xyloside could induce an increase in DS, and that DS can enable enhanced function of FGF-10, we next hypothesized that C-Xyloside might increase NHEK responsiveness to FGF-10, thus providing a potential explanation for the effects observed *in vivo*. Treatment with a combination of both C-Xyloside and FGF-10 enhanced NHEK migration compared to treatment with C-Xyloside or FGF-10 alone (Figure 4A, B). This effect was similar to results seen following stimulation with exogenously added DS plus FGF-10, a treatment previously shown to enhance keratinocyte migration.

### Conditioned media from keratinocytes treated with C-Xyloside facilitates FGF-10-dependent cell proliferation

Since BaF3/KGFR cells express FGFR2-IIIb constitutively and proliferate in a FGFR2-IIIb-dependent manner, BaF3/KGFR cells have been used as a model system to specifically study the interaction between FGFR2-IIIb and FGF-10. C-Xyloside (1 mM) itself did not facilitate BaF3/KGFR cell proliferation in the presence of FGF-10 (data not shown). Conditioned media from keratinocytes treated with or without C-Xyloside was then used to stimulate BaF/KGFR cells in the presence of FGF-10. Media from keratinocytes treated with C-Xyloside enhanced BaF3/KGFR cell proliferation only in the presence of FGF-10, and similar to the enhancement seen with DS (Figure 4C). These results further support observations that NHEK treated with C-Xyloside releases DS and has functional significance in terms of growth factor response.

## Discussion

In the present study, we show that a C-xylopyranoside derivative can act as a potent stimulus for increased synthesis of DS by keratinocytes and enables enhanced FGF-10 responsiveness. This is a potentially critical developmental function as FGF-10 is essential for epithelial development and wound repair. This was clearly shown in results from FGFR2-IIIb null mice which exhibit severe developmental malformations in cardiac [17], limb [18], bone [19], lung, tooth, and thymic [20] development. These mice also display severe epidermal hypoplasia within basal keratinocytes, demonstrating the important role for this receptor, and its ligand FGF-10, in keratinocyte migration, proliferation, and differentiation [21].

The function of FGF-10 is dependent, at least in part, upon cutaneous DS produced during injury [11]. The purpose of this study was to test the hypothesis that a C-xylopyranoside derivative could induce the production of GAGs such as DS from human keratinocytes, and thus influence functions dependent on DS. Measurement of soluble GAGs in culture media from keratinocytes demonstrated that large amounts of sulfated GAGs were present in the media after C-Xyloside treatment. This GAG was not heparan sulfate since most of the soluble GAG was digested only by chondroitinase ABC. Furthermore, the GAGs synthesized by C-Xyloside addition included IdoA residues and were highly sulfated, containing more than 35% of D0a4. This indicated that the newly synthesized GAG was DS and contained the specific sulfation which has optimal activity for enabling FGF function [2]. Therefore, although we can not exclude the possibility that some additional HS and CS was produced by these cells, a large proportion of the total GAG released by NHEK was probably a biologically active form of DS.

To confirm that the DS produced was indeed biologically active, we performed an *in vitro* scratch assay. This showed that C-Xyloside augmented the ability of keratinocytes to migrate, a critical process required for optimal wound repair, and a process also enhanced by DS. Furthermore, by using FGFR2-IIIb expressing cells, we showed that C-Xyloside itself did not facilitate cell proliferation, nor did it induce growth factor release, but rather induced a molecule that could only stimulate proliferation in the presence of FGF-10. Combined, this is strongly suggestive that C-Xyloside treatment of keratinocytes results in production of DS, and that this facilitates the action of FGF-10.

The significance of the current observations to skin biology is several-fold. First, we show here for the first time a biochemical strategy to alter GAG synthesis by keratinocytes. As GAGs are critical components of the extracellular matrix of the skin, and these molecules function in a variety of ways that range from establishing structural integrity to modifying growth factor and enzymatic function, a simple and direct system to enhance GAG synthesis is an exciting development. Furthermore, we show that under the experimental conditions used here, keratinocytes appear primed to initiate DS above other GAGs. This supports prior work suggesting DS has unique importance in the skin and may in fact be more relevant to growth factor responses than the much more widely studied GAG HS. Thus, in future studies it would be of interest to evaluate *in vivo* the responses to topical application C-Xyloside to determine if this might augment the activity of growth factors such as FGF-10 to promote wound repair or enhance epidermal growth. This novel approach suggests a strategy to enhance endogenous growth factor activity in abnormal wound repair without the limiting expense of delivery of excess recombinant proteins.

## Materials and Methods

### Reagents

DS from porcine intestinal mucosa (DS-03122) was purchased from Celsus Laboratories Inc. (Cincinnati, OH). Chondroitinase ABC (C-3667) were purchased from Sigma (St. Louis, MO). Recombinant FGF-10 was purchased from R&D Systems (Minneapolis, MN). C-β-D-xylopyranoside-2-hydroxy-propane (C-Xyloside) was synthesized by L'Oréal Research Laboratories (France).

### Cell culture

Normal human epidermal keratinocytes (Invitrogen, Gibco Cell Culture, Portland, OR) were grown to 80% confluence in Epilife® medium containing 0.06 mM Ca^2+^, Epilife defined growth supplement (EDGS) (Invitrogen, Gibco Cell Culture), 50 U/ml penicillin, and 50 µg/ml streptomycin at 37°C in a humidified atmosphere of 5% CO_2_. The human primary foreskin fibroblasts cell line 12F were provided by Advanced Tissue sciences (La Jolla, CA) and were grown in Dulbecco's modified Eagle's medium (DMEM; Gibco BRL, Rockville, MD) supplemented with 10% fetal bovine serum (HyClone, Logan, UT), L-glutamine, penicillin and streptomycin. CHO-K1 cell lines were obtained from American Type Culture Collection (Manassas, VA). A mutant CHO cell line pgsA-745 (designated here as CHO-745) [13] were kind gift from Prof. J.D. Esko (University of California, San Diego, CA).

### Cell treatment with C-Xyloside, and glycosaminoglycan quantitation

Cells, grown to 80% confluence in media, were incubated with 0, 0.3, 1 mM of C-Xyloside for 24 or 48 hours. The cultured media were harvested. Sulfated GAG in the form of PG or free GAG chains was purified from the cell lysates as previously described [1]. Total sulfated PG/GAG was quantified using Blyscan assay according to the manufacturer's instructions (Accurate Chemical Science Corp., Westbury, NY). Blyscan assay is a quantitative dye-binding method for analysis of sulfated GAGs.

### Disaccharide Analysis and Uronic acid determination, HPAEC-PAD

The Glycotechnology Core at the University of California, San Diego, performed all disaccharide analyses. For uronic acid determination, the GAG samples were treated with 2 M TFA at 100°C for 6 hours to cleave all glycosidic linkages. After drying the hydrolyzate, samples were dissolved in water and analyzed by HPAEC-PAD using a CarboPac PA-1 column. Common uronic acid standards (Glucuronic, Galacturonic and iduronic acids) were treated in parallel and used for calibration of HPAEC-PAD response.

For disaccharide analysis, the GAG samples were digested completely using chondroitinase ABC. The analysis and separation of disaccharides were achieved by using reverse phase ion pair chromatography (C18 TosoHaas ODS-120T column, 4 um particle size), with post-column derivation based on the method described previously by Sakai et al [22]. We also carried out Mass spectrometry using glycan reductive isotope labeling as described [15]. Briefly disaccharides were labeled with [^12^C]aniline. The labeled mixture of disaccharides was combined with 25 pmol each of disaccharide standards labeled with [^13^C]aniline, and the sample was subjected to mass spectrometry. Radiometric analysis of the 12C-and 13C-labeled disaccharides provided a quantitative way to determine the level of each disaccharide.

### *In vitro* keratinocyte wound assay

The capacity of normal human keratinocytes to migrate, and close a defined area of injury to a confluent monolayer *in vitro* was evaluated by a previously reported assay [11], [23]. Primary human keratinocytes (NHEK) were stimulated with DS (2 µM) alone, FGF-10 (10 ng/mL) alone, C-Xyloside (1 mM) alone, DS and FGF-10, C-Xyloside and FGF-10. PBS was used as a vehicle control (untreated cells). The same fields were photographed every 24 hours, and the area migrated over by keratinocytes was calculated using Image J Software (NIH Image). The percent migration at 24 hours was calculated by subtracting the total area of the scratched at 24 hours from the total area at 0 hour, divided by the total area at 0 hour multiplied by 100. Images of the areas were collected with an Olympus MVX10 microscope equipped with a DC71 camera (Olympus, Center Valley, PA). This experiment was repeated three times, and the mean of all three experiments are shown.

### Cellular proliferation assay

BaF/KGFR cells were selected as previously described [7]. To collect cultured media, primary keratinocytes were treated with or without C-Xyloside (1 mM) in RPMI1640 for 24 hours. All dilutions of GAG preparations and cells were done in this keratinocyte-conditioned medium. Before experiments, BaF/KGFR cells were washed three times in PBS before seeding cells at a final concentration of 10000 cells/well. Experiments were conducted in 96-well plates (Costar, Fisher Scientific, Pittsburgh, PA) with a final volume of 100 µl/well. FGF-10 were used at a final concentration of 5 ng/ml. Cells were incubated for 3 days at 37°C before performing a nonradioactive cell proliferation assay (MTT assay, Chemicon® international, Millipore, Billerica, MA).

### Statistical analysis

One-way or Two-way ANOVA was used to determine significance in the experiments *in vitro*, which were analyzed by GraphPad Prism 4 (GraphPad Software, Inc.).
